# Editorial: Spontaneous Activity in Sensory Systems

**DOI:** 10.3389/fncir.2018.00027

**Published:** 2018-03-29

**Authors:** Kazuo Imaizumi, Edward S. Ruthazer, Jason N. MacLean, Charles C. Lee

**Affiliations:** ^1^Wyss Institute for Biologically Inspired Engineering, Harvard University, Boston, MA, United States; ^2^Department of Neurology and Neurosurgery, Montreal Neurological Institute, McGill University, Montreal, QC, Canada; ^3^Department of Neurobiology, The University of Chicago, Chicago, IL, United States; ^4^Department of Comparative Biomedical Sciences, LSU School of Veterinary Medicine, Baton Rouge, LA, United States

**Keywords:** visual system, auditory system, somatosensory system, tinnitus, retina, lateral geniculate nucleus (LGN), cerebral cortex, computational neuroscience

Spontaneous activity in the nervous system, particularly in sensory systems, has become increasingly appreciated due to its important impact on the developing and mature brain. Although by its nature, spontaneous activity has been difficult to study experimentally, recent advances have shed light on its unique function in patterning the nervous system and information processing in normal and diseased conditions of the adult brain. In this special research topic for *Frontiers in Neural Circuits*, we bring together a collection of work from experts in the field that both summarizes past discoveries and introduces current advances to our understanding of spontaneous activity in both developing and mature sensory systems. Here, we provide a summary of their contributions.

## Developing sensory systems

Among all of the sensory systems, spontaneous activity is perhaps first and most intensively investigated in the developing visual system, extending from local propagating activity in the retina to central visual pathways. In this regard, Kerschensteiner provides a review of the patterns, mechanisms, and functions of glutamatergic retinal waves, particularly their suitability for refining physiological properties in the visual thalamus (lateral geniculate nucleus: LGN) and the primary visual cortex (V1). The spatiotemporal properties of these retinal waves are further considered by Arroyo and Feller, who discuss the role of inter-eye competition and intraretinal correlated activity in the development of eye-specific segregation and retinotopy in the visual system.

The development of central visual structures is considered in more detail by a group of articles that focus on the projections from the retina to the optic tectum (the retinotectal projection) in tadpoles. In their article, Kutsarova et al. posit a set of rules underlying the development of the retinotectal pathway, which encompass a range of molecular, synaptic, physiological and homeostatic mechanisms. The tectothalamic pathway is further explored by Jang et al., who describe their computational network model of selective visual responses in the optic tectum to looming stimuli, which is able to predict simple behavioral responses in tadpoles. Finally, Pratt et al. examine the role of retinal waves and the development of the retinotectal projections in an evolutionary context, providing predictions for studies of retinal projections in mammalian systems to the superior colliculus (SC) and LGN.

Of course, spontaneous activity plays a key role in the development of all sensory systems. In their article, Leighton and Lohman provide an extensive review of the role of spontaneous activity in the wiring of the developing auditory, somatosensory and visual systems of rodents, and further integrate these with synaptic plasticity mechanisms. Furthermore, the development of higher order structures in each sensory system, particularly the cerebral cortex, is influenced by spontaneous neural activity. Luhman et al. discuss the role of spontaneous activity patterns in the development of neocortical networks and how these arise from changes in the biophysical properties of single neurons to the eventual involvement of complex network interactions. They suggest that early disturbance to these activity patterns may produce permanent neural alterations. Such early-life perturbations are examined by Shoykhet and Middleton, who describe alterations to spontaneous activity in the developing somatosensory and motor system of the rat thalamocortical system, following cardiac arrest-induced global brain hypoxia-ischemia, and they suggest that modulation of the abnormally induced spontaneous activity patterns could improve functions in cardiac arrest survivors.

## Mature sensory systems

In mature sensory systems, spontaneous activity underlies both normal and diseased processing of information. In this context, the mature neocortex has been the focus of most investigations of the role of spontaneous activity in shaping and refining sensory information processing. In his review, Tan examines the diversity of such spontaneous activity across the sensory neocortex, highlighting both similarities and differences across cortical layers and areas, during fixation and slow oscillations, and the effects of attention, anesthesia and plasticity. Such activity patterns, may reveal facets of the diversity of functional properties across different regions of the neocortex. The role of behavioral state is further explored by McVea et al., who examine and compare the role of slow-wave activity in the adult brain with spindle-burst-related spontaneous activity in the developing brain. They note that both patterns of spontaneous activity have important, but likely independent, functional roles in developing and mature sensory systems.

The final set of articles examine the role of spontaneous activity in diseased sensory systems, in particular the auditory system. An important disorder here is tinnitus, which is the phantom perception of sound, largely thought to originate from excessive spontaneous activity throughout the auditory pathway. Chen et al. present evidence consistent with this view in their fMRI study of individuals with chronic tinnitus. Their study describes alterations in low-frequency fluctuations in several auditory and non-auditory neural centers of those with tinnitus. In an alternate view, Eggermont develops the notion that spontaneous activity is an information carrier, via firing rate and neural synchrony, throughout the auditory pathway, rather than a source of noise. In this context, he argues that increased spontaneous activity during tinnitus alters the activity of both auditory and non-auditory neural structures, which in turn results in the tinnitus percept.

## Conclusion

As can be appreciated, the articles in this special topic on spontaneous activity in sensory systems cover a wide-range of advances in our understanding of its role in developing and mature nervous systems. However, these articles should also highlight avenues for future investigation, particularly as they pertain to the growing ability to manipulate neural activity to restore normal sensory function following aberrant development or in diseased states.

## Author contributions

KI, ER, JM, and CL all contributed to the inception, solicitation, drafting and editing of this special topic and editorial.

### Conflict of interest statement

The authors declare that the research was conducted in the absence of any commercial or financial relationships that could be construed as a potential conflict of interest.

